# Distribution of lipid levels and prevalence of hyperlipidemia: data from the NHANES 2007–2018

**DOI:** 10.1186/s12944-022-01721-y

**Published:** 2022-10-28

**Authors:** Zhenhan Li, Guoqi Zhu, Guo Chen, Mei Luo, Xuebo Liu, Zhongpei Chen, Jun Qian

**Affiliations:** 1 Department of Endocrinology, Chongqing Hospital Of Traditional Chinese Medicine, Chongqing, China; 2 Department of Cardiology, Tongji Hospital, Tongji University School of Medicine, Shanghai, China

**Keywords:** Hyperlipidemia, Lipid levels, Age, Sex, Race, Smoking status, NHANES database

## Abstract

**Background:**

Lipid-lowering therapy is important, and the distribution of lipid levels and the incidence of hyperlipidemia may vary in different subgroups of the population. We aimed to explore the distribution of lipid levels and the prevalence of hyperlipidemia in subpopulations with subgroup factors, including age, sex, race, and smoking status.

**Methods:**

Our study used data from the National Health and Nutrition Examination Survey (NHANES) from 2007 to 2018, ultimately enrolling and analyzing 15,499 participants. A cross-sectional analysis was performed to assess the distribution of lipids and prevalence of hyperlipidemia in subpopulations, and multifactorial logistic regression analyses were performed for the prevalence of hyperlipidemia, adjusted for age, sex, race and smoking status.

**Results:**

Blacks had significantly lower mean serum total cholesterol and triglycerides and higher serum high-density lipoprotein cholesterol (HDL-C) than whites (*P* < 0.001). In contrast, Mexican Americans had markedly higher mean serum triglycerides and lower serum HDL-C than whites (*P* < 0.001). Furthermore, the prevalence of hypercholesterolemia and hypertriglyceridemia was lower in blacks than in whites (*P* = 0.003 and *P* < 0.001, respectively), while the prevalence of hypertriglyceridemia was significantly higher in Mexican Americans than in whites (*P* = 0.002). In addition, total cholesterol and triglyceride levels were significantly higher in women aged 65 years or older and markedly higher than in men in the same age group (*P* < 0.001). In addition, overall mean total cholesterol, triglyceride, and low-density lipoprotein cholesterol (LDL-C) levels were higher in smokers than in nonsmokers (*P* = 0.01, *P* < 0.001, and *P* = 0.005, respectively).

**Conclusion:**

Based on NHANES data, the mean lipid levels and prevalence of hyperlipidemia differed by sex, age, race, and smoking status.

## Background

Hyperlipidemia is defined as a higher-than-normal level of one or more lipids in plasma, which is clinically classified as hypercholesterolemia, hypertriglyceridemia, mixed hyperlipidemia, and high-density lipoproteinemia in the clinic [1, 2]. Epidemiological studies and clinical trials have confirmed that elevated low-density lipoprotein cholesterol (LDL-C), cholesterol, or triglycerides are the major contributors to atherosclerotic cardiovascular disease [3, 4]. Generally, the risk of atherosclerotic events increased significantly when plasma total cholesterol levels exceeded 240 mg/dl or triglyceride levels exceeded 200 mg/dl [5, 6]. Furthermore, hyperlipidemia-induced atherosclerosis increases morbidity and mortality in a quiet modality, including myocardial infarction, cerebral infarction, and peripheral vascular disease [4]. In patients with hyperlipidemia, the incidence of cardiovascular events remains high even when treated with secondary or primary prevention therapy, such as statins, where elevated triglyceride levels can serve as an independent marker of increased risk of ischemic events [7]. In the United States (US), Canadian and European guidelines for the management of hypercholesterolemia, LDL-C is the primary target for lipid-lowering therapy, based on which hypercholesterolemia is the most important pathogenic factor in atherosclerotic cardiovascular disease [8].

The American Heart Association Task Force on Clinical Practice Guidelines proposed individualized management and lifestyle interventions that consider risk factors while lowering LDL-C with high-intensity statins with reference LDL-C thresholds, including 70 mg/dL (1.8 mmol/L) and 100 mg/dL (2.6 mmol/L), and age points of 40 and 75 years [9]. The guidelines related to hyperlipidemia are graded as low risk, moderate risk, moderate-high risk, and high risk in that order based on a comprehensive assessment of risk factors (e.g., sex, age, smoking, hypertension, coronary heart disease, etc.), cholesterol and triglyceride levels [9, 10]. In addition, the corresponding treatment is administered according to the risk level after hyperlipidemia assessment to reduce the occurrence of cardiovascular events and other complications [11, 12]. However, race/ethnicity can influence the estimation of atherosclerotic cardiovascular disease risk, treatment intensity, and even the use of lipid-lowering drugs, such as the higher risk of atherosclerotic cardiovascular disease in South Asians, greater statin sensitivity in East Asians, and higher prevalence of hypertension in blacks [13]. Therefore, the specific distribution of blood lipid levels by race, sex, and age is still unclear, and current treatments based on risk stratification of lipids also do not consider differences in those factors [14, 15].

The objective of our study was to investigate the distribution of blood lipid levels by race, sex, age, and smoking status in the population based on data from the National Health and Nutrition Examination Survey (NHANES): 2007–2018. These data will refine lipid-related information for different subpopulations and provide a reference for more accurate cardiovascular event prevention treatment in different populations.

## Methods

### Data collection

NHANES is a representative series of cross-sectional studies designed to monitor the health status of the US population. NHANES data consist of interviews, examinations, and laboratory data collected from a complex, multistage, stratified, aggregated probability sample of civilians and nonhospital personnel [16]. The institutional Review Board of the National Center for Health Statistics (NCHS) approved the study protocol, and all participants provided written informed consent. In NHANES from 1999 to 2018, participation in-home interviews was 52–84%, and the percentage in mobile medical examinations was 49–80%. Our study included adult participants aged 18 years or older in NHANES from 2007 to 2018. The exclusion criteria included the following: (1) participants under the age of 18; (2) participants who did not attend screening centers; and (3) participants with missing lipid values.

### Sociodemographic characteristics, laboratory testing, and definition

Participants reported age, sex (male or female), race (white, black, Mexican-American), and smoking status (smoker or nonsmoker). NHANES organizers collected 3 ml or 5 ml of K3 EDTA anticoagulant whole blood from all participants 18 years of age or older using established venipuncture protocols and procedures. Total cholesterol, triglycerides, LDL-C, and serum high-density lipoprotein cholesterol (HDL-C) values were measured enzymatically. Hypercholesterolemia was defined as fasting total cholesterol values ≥ 240 mg/dl. High triglycerides were defined as fasting triglyceride values ≥ 200 ng/dl. Smokers were defined as smoking more than 100 cigarettes in life and smoking some days or every day, while nonsmokers smoked less than 100 cigarettes in life.

### Statistical analysis

Our analyses used a weighted sample and took into account stratification and clustering designed to derive estimates of the U.S. population [17]. All statistical analyses were carried out using R software (version 4.1.0; http://www.R-project.org, R Foundation for Statistical Computing, Vienna, Austria). The unadjusted frequency distribution of blood lipid values and unadjusted prevalence of hyperlipidemia associated with age, sex, race, and smoking status were identified. We used linear regression to obtain mean values and comparisons of age-adjusted and sex-adjusted blood lipids, where age was modeled as a continuous variable. We also compared the adjusted mean blood lipids of smokers and nonsmokers. Multivariate logistic regression analysis was performed for the prevalence of hypercholesterolemia and hyperlipidemia, adjusting for age, sex, and ethnic groups. More complex models including ethnicity and sex were also tested, and different age classifications were considered.

## Results

### Mean blood lipid values among sex, age, and ethnic group

**Fig. 1:**
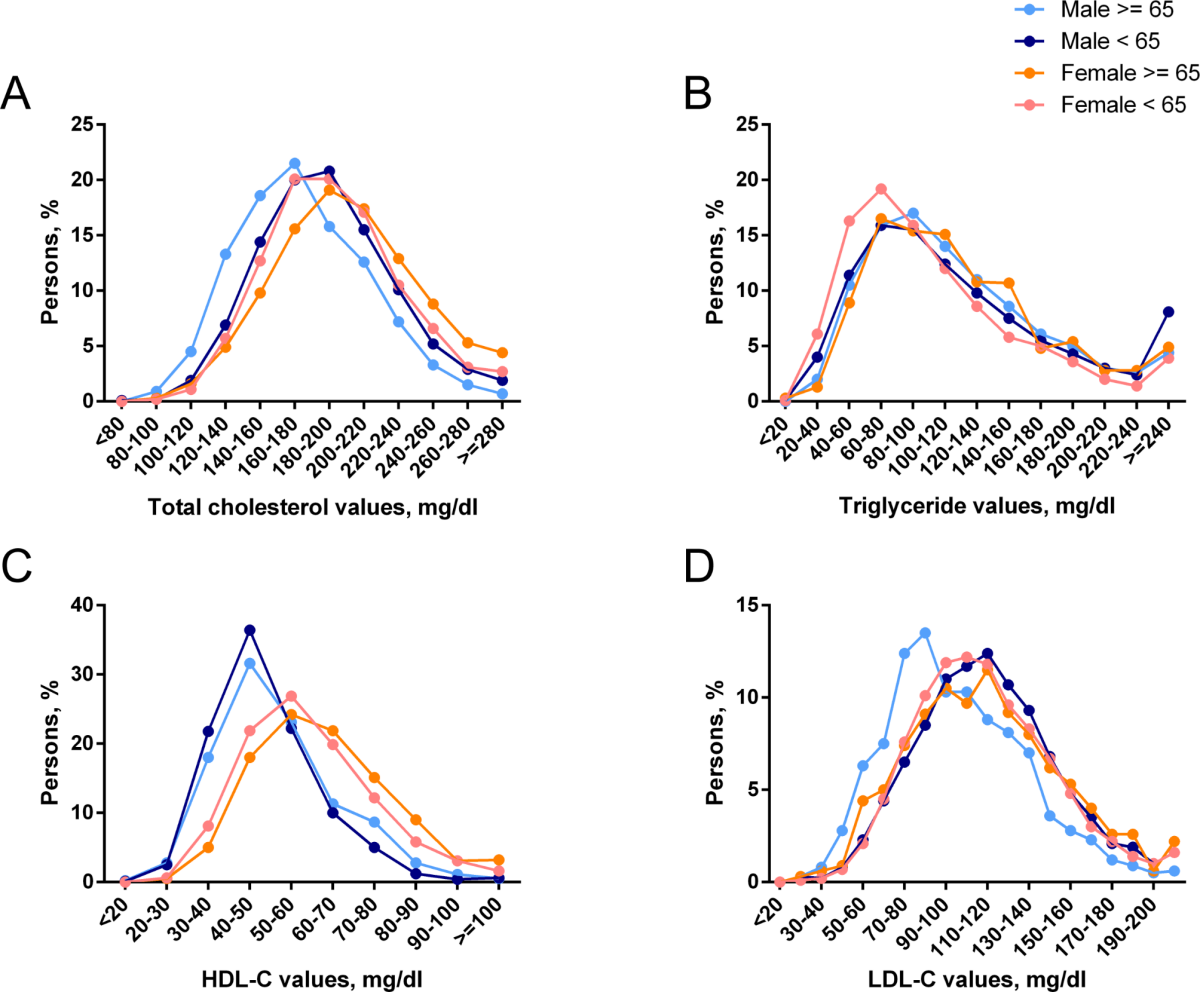
The actual percentage and number of participants with different mean lipid values based on gender and age

Of the 36,580 participants aged 18 or older who were asked to attend the mobile examination center, 1,418 did not come to the screening center, and 19,663 had missing blood lipid values. A total of 15,499 NHANES participants were enrolled in the study, which represented 104.4 million United States residents. We used linear regression to compare age-adjusted and sex-adjusted mean blood lipid values across the major races. Relative to white participants, black participants had lower mean total cholesterol levels (mean difference, 4.0 mg/dl; *P* < 0.001), lower triglyceride levels (24.7 mg/dl; *P* < 0.001), higher HDL-C levels (2.2 mg/dl; *P* < 0.001), and similar LDL-C levels (1.3 mg/dl; *P* = 0.19). Mexican-American participants had similar mean total cholesterol levels (0.9 mg/dl; *P* = 0.47), higher triglyceride levels (11.9 mg/dl; *P* < 0.001), lower HDL-C levels (3.0 mg/dl; *P* < 0.001), and similar LDL-C levels (1.5 mg/dl; *P* = 0.21) compared with white participants **(**Table 1**)**. Total cholesterol, triglyceride and HDL-C levels in females aged 65 years or older were significantly higher than those in females aged less than 65 years, while the opposite trend was observed in males except HDL-C (*P* < 0.05). In addition, we observed that in the population aged 65 years or older, females had higher cholesterol, triglyceride, HDL-C and LDL-C levels than males, except Mexican-American participants (*P* < 0.05). In addition, the triglyceride levels of females were lower than those of males in the population aged less than 65 years, while the trend was the opposite for LDL-C (*P* < 0.05) **(**Fig. 1; Table 2**)**.

**Table 1:** Age-adjusted and sex-adjusted mean blood lipid levels by using linear regression

	Mean Value	Mean Difference	*P* value
**Total cholesterol**			
Whites	191.6 (190.1-193.1)	Ref.	/
Blacks	186.2 (184.6-187.9)	4.0 (2.0-5.9)	< 0.001
Mexicans	189.0 (186.7-191.4)	0.9 (-1.6-3.5)	0.47
**Triglyceride**			
Whites	117.4 (114.9-119.8)	Ref.	/
Blacks	89.2 (86.7–91.7)	24.7 (21.6–27.8)	< 0.001
Mexicans	124.8 (121.2-128.4)	11.9 (7.6–16.2)	< 0.001
**HDL-C**			
Whites	54.9 (54.3–55.5)	Ref.	
Blacks	57.2 (56.5–57.9)	2.2 (0.8–3.6)	< 0.001
Mexicans	50.9 (50.2–51.6)	3.0 (2.0–4.0)	< 0.001
**LDL-C**			
Whites	113.3 (112.1-114.5)	Ref.	
Blacks	111.2 (109.5-112.8)	1.3 (-0.5-3.1)	0.19
Mexicans	113.2 (111.1-115.3)	1.5 (-0.7-3.7)	0.21

Means (95% CIs) are given from a weighted analysis adjusted for age and sex. Abbreviations: HDL-C, high density lipoprotein cholesterol, LDL-C, low density lipoprotein cholesterol, Ref, Reference

**Table 2:** Mean blood lipid values, by age, sex, and ethnic group

	Whites	Blacks	Mexicans
Total cholesterol			
Males < 65y	188.1(185.9-190.3)	185.9 (183.4-188.5)	191.5 (188.4-194.7)
Males ≥ 65y	174.9(171.8-178.1)‡	173.3 (168.5-178.1) ^‡^	177.7 (171.6-183.7) ^‡^
Females < 65y	195.9(194.0-197.7)	186.5 (184.1-188.8)	187.1 (184.1-190.2)
Females ≥ 65y	202.8(199.3-206.3)#§	194.7 (188.8-200.7) ^#§^	189.9 (185.6-194.2) ^#§^
Triglyceride			
Males < 65y	123.7(120.1-127.4)	98.9 (95.1-102.6)	133.9 (129.0-138.8)
Males ≥ 65y	120.1(115.0-125.2)‡	91.9 (86.0- 97.9) ^‡^	127.4 (114.9–140.0) ^‡^
Females < 65y	108.3(104.7-111.8)¶	81.1 (78.5–83.8)	113.5 (109.4-117.7)
Females ≥ 65y	123.4(118.5-128.3)#§	90.3 (84.9–95.7) ^#§^	135.2 (126.4–144.0) ^#§^
HDL-C			
Males < 65y	48.5(47.8–49.2)	53.1 (52.2–54.0)	47.2 (46.3–48.1)
Males ≥ 65y	51.0(49.4–52.6)‡	54.9 (53.2–56.5) ^‡^	50.1 (47.2–53.1) ^‡^
Females < 65y	59.4(58.5–60.4)¶	59.7 (58.6–60.8)	54.3 (53.4–55.1)
Females ≥ 65y	63.5(61.8–65.1)#§	63.6 (61.6–65.7) ^#^	57.4 (55.1–59.7) ^#^
LDL-C			
Males < 65y	114.8(113.0-116.6)	113.1 (110.7-115.5)	117.5 (114.7-120.4)
Males ≥ 65y	100.0(97.2-102.7)‡	100.0 (95.7-104.3)	102.1 (96.8-107.4)
Females < 65y	114.8(113.3-116.2)	110.5 (108.4-112.7)	110.1 (107.4-112.9)
Females ≥ 65y	114.6(111.5-117.7)§	113.0 (107.3-118.8)	105.5 (101.5-109.4)

Means (95% CIs) are given from a weighted analysis adjusted for age and sex

^#^, *P* < 0.05 Females ≥ 65y compared with females < 65 y

^‡^, *P* < 0.05 Males ≥ 65y compared with males < 65 y

^¶^, *P* < 0.05 Females < 65y compared with males < 65 y

^§^, *P* < 0.05 Females ≥ 65y compared with males ≥ 65 y

### Mean blood lipid values among the smoker and nonsmoker groups

We compared mean age-adjusted and sex-adjusted blood lipid levels between smokers and nonsmokers **(**Table 3**)**. Smokers had higher overall mean total cholesterol, triglycerides, and LDL-C levels than nonsmokers (*P* = 0.01, *P* < 0.001, *P* = 0.005, respectively). However, there was no significant difference in total cholesterol levels among ethnic groups whether they smoked or not (*P* = 0.06 for whites, *P* = 0.32 for blacks, and *P* = 0.17 for Mexican-Americans). We also found that smokers had lower overall mean HDL-C levels than nonsmokers, which was consistent in white participants (all *P* < 0.001). Smoking had the greatest effect on the increase in triglyceride levels among Mexican-Americans (17.2 mg/dL), the smaller effect in white participants (14.7 mg/dL), and the smallest effect in black participants (12.8 mg/dL).

**Table 3:** Comparative Mean Blood Lipid Values in Smokers and Non-smokers

Variable	Smokes	Nonsmokers	Mean Difference between Smokers and Nonsmokers^*^	*P* value^†^
**Total cholesterol**				
All	191.6(189.3,193.8)	190.8(189.7,192.0)	2.9(0.5–5.3)	0.01
Whites	192.5(189.4,195.5)	191.8(190.3,193.3)	3.1(-0.03-6.2)	0.06
Blacks	184.8(181.6,188.1)	187.5(185.5,189.6)	1.9(-1.8,5.6)	0.32
Mexicans	192.7(187.7,197.7)	189.2(186.9,191.6)	3.2(-1.3-7.8)	0.17
**Triglyceride**				
All	125.1(121.1,129.1)	113.0(111.0,115.0)	13.6(9.5–17.7)	< 0.001
Whites	127.0(122.0,132.1)	115.4(112.9,117.9)	14.7(9.4–20.0)	< 0.001
Blacks	100.4(96.0,104.8)	85.8(82.9, 88.7)	12.8(7.7–17.8)	< 0.001
Mexicans	142.4(132.2,152.7)	122.5(118.7,126.4)	17.2(6.3–28.0)	< 0.001
**HDL-C**				
All	51.5(50.7,52.4)	55.3(54.8,55.8)	2.6(1.8–3.4)	< 0.001
Whites	51.4(50.3,52.5)	55.7(55.1,56.4)	3.4(2.3, 4.6)	< 0.001
Blacks	56.0(54.6,57.4)	57.7(56.9,58.5)	0.5(-1.0,2.1)	0.51
Mexicans	48.1(46.5,49.8)	51.3(50.6,52.1)	1.8(-0.2,3.7)	0.08
**LDL-C**				
All	115.0(113.2,116.9)	112.9(112.0,113.9)	2.8(0.8–4.8)	0.005
Whites	115.6(113.1,118.2)	113.0(111.7,114.2)	3.6(-0.8-6.4)	0.01
Blacks	108.8(105.6,112.0)	112.7(110.8,114.6)	3.9(0.3,7.6)	0.04
Mexicans	116.1(112.0,120.1)	113.4(111.2,115.5)	1.5(-2.2-5.3)	0.43

^*^ The respective numbers of participants who smoked and those who did not smoke were 757 and 2296 for black- 1356 and 4847 for white- and 331 and 1990 for Mexican-American participants

^†^ Comparisons of the means between smokers and nonsmokers- according to ethnicity and adjusted for age and sex

### Prevalence of hyperlipemia in different sexes, ages, and ethnic groups

**Fig. 2:**
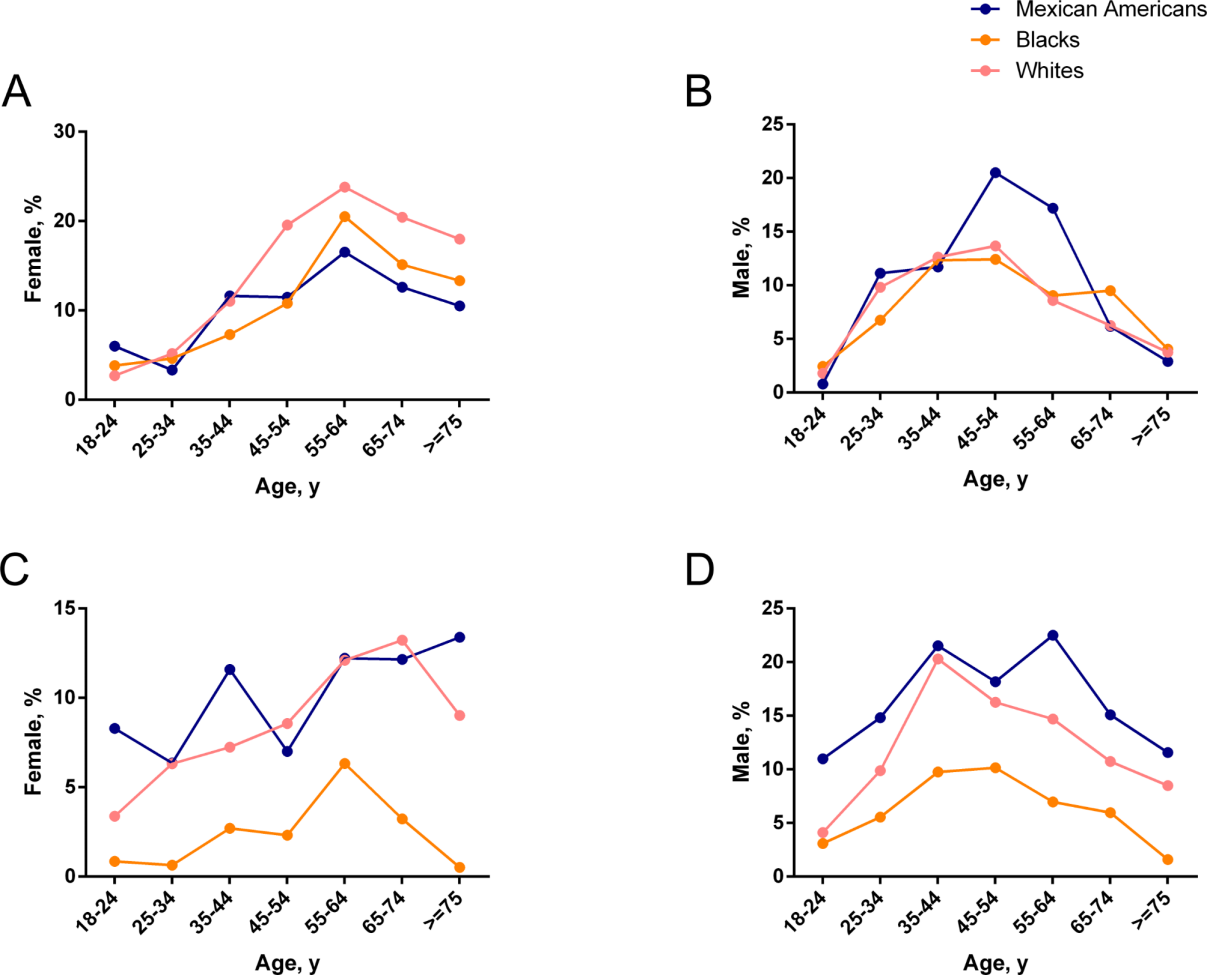
The actual percentage and number of participants with different incidences of hyperlipidemia based on race and age

Hypercholesterolemia (total cholesterol values ≥ 240 mg/dl) and hypertriglyceridemia (triglyceride levels ≥ 200 mg/dl) differed by sex, age, and ethnic group **(**Fig. 2; Table 4**)**. A total of 1,715 and 1,616 participants had hypercholesterolemia and hypertriglyceridemia, with a weighted prevalence of 11.5% (95% CI, 10.6–12.5%) and 10.4% (CI, 9.4–11.4%), representing an estimated 12.05 million and 10.84 million Americans, respectively. Hypercholesterolemia and hypertriglyceridemia were present in 12.2% (CI, 11.3–13.1%) and 10.9% (CI, 9.8–11.9%) of white participants, 9.4% (CI, 8.3–10.4%) and 4.4% (5.1% CI, 3.7%) of black participants, and 10.1% (CI, 8.5–11.7%) and 13.2% (CI, 11.5–14.5%) of Mexican-American participants **(**Table 4**)**. Among ethnic groups, males were more likely to have hypertriglyceridemia: white males 13.0% versus white females 8.9%, black males 6.9% versus black females 2.5%, Mexicans-American males 14.3% versus Mexicans-American females 9.2%. Nevertheless, the proportion of females with hypercholesterolemia was higher than that of males. In every age and sex category, black participants were less likely to have hypertriglyceridemia than white participants, and hypertriglyceridemia was more common among Mexican-American participants than among white participants.

**Table 4:** Prevalence of hypercholesteremia and hypertriglyceridemia

Variable	Hypercholesteremia Prevalence-%^×^(95% CI)	*P* value	Hypertriglyceridemia Prevalence-%^×^(95% CI)	*P* value
**Ethnicity**				
Whites	12.2(11.3–13.1)	Ref.	10.9(9.8–11.9)	Ref.
Blacks	9.4(8.3–10.4)	0.003	4.4(3.7–5.1)	< 0.001
Mexicans	10.1(8.5–11.7)	0.70	13.2(11.5–14.5)	0.002
**Sex**				
Female	13.7(12.8–14.6)	Ref.	8.0(7.0–9.0)	Ref.
Male	9.2(8.4–10.1)	< 0.001	12.9(11.9–13.9)	< 0.001
**Age**				
18–24	2.8(1.7–4.01)	Ref.	4.6(3.5–5.8)	Ref.
25–34	7.1(5.8–8.3)	< 0.001	7.8(6.5–9.1)	< 0.001
35–44	11.3(9.5–13.1)	< 0.001	12.9(11.2–14.7)	< 0.001
45–54	15.9(13.8–18.1)	< 0.001	11.9(10.3–13.5)	< 0.001
55–64	16.1(14.1–18.0)	< 0.001	12.6(10.5–14.7)	< 0.001
65–74	13.4(11.2–15.5)	< 0.001	11.4(9.2–13.6)	< 0.001
≥ 75	11.7(9.8–13.6)	< 0.001	8.6(7.1–10.0)	< 0.001
**Smoke**				
Yes	12.8(11.2–14.3)	Ref.	13.4(11.4–15.3)	Ref.
No	11.4(10.7–12.2)	0.002	9.7(9.0-10.5)	< 0.001

^**×**^Prevalence is estimated as the predicted marginals in the logistic regression analysis. The estimates for white- black- and Mexican-American participants are adjusted for age and sex

Multivariate logistic regression analysis was performed for the prevalence of hyperlipidemia, adjusted for sex, age, and race **(**Table 4**)**. Black participants were less likely to have hypercholesterolemia (*P* = 0.003) or hypertriglyceridemia (*P* < 0.001) than white participants. Mexican-Americans had no significant difference in the prevalence of hypercholesterolemia (*P* = 0.70) but had a significantly higher prevalence of hypertriglyceridemia (*P* = 0.002) than white participants. Males had less hypercholesterolemia (*P* < 0.001) but more hypertriglyceridemia (*P* < 0.001) than females. The prevalence of hyperlipidemia peaked from the age of 35 to 65 years and generally decreased after the age of 65 years. In independent logistic regression analysis adjusted for sex, age, and race, smokers had significantly higher percentages of hypercholesterolemia (*P* = 0.002) and hypertriglyceridemia (*P* < 0.001) than nonsmokers.

## Discussion

In this analysis of nationally representative data, the mean lipid levels and prevalence of hyperlipidemia were different among sex, age, smoking status, and ethnic group. The mean values of serum total cholesterol and triglycerides in black participants were significantly lower than those in white participants, and serum HDL-C was higher than in white participants. Mexicans-American participants had considerably higher average serum triglycerides and lower serum HDL-C than white participants. Black participants had a notably lower prevalence of hypercholesterolemia or hypertriglyceridemia than white participants, and Mexican-American participants had a significantly higher prevalence of hypertriglyceridemia than white participants. Similarly, a CARDIA study of long-term trends in lipid levels in whites and blacks, men and women in four U.S. communities followed by a multifactorial regression stratified by race and sex analysis found that LHD-C increased with age, except for black women who consistently maintained lower LHD-C levels, while triglycerides showed an unfavorable long-term upward trend in white women [18]. Studies on the association between age and lipid levels are still controversial, for example, in Spain, France, Finland, and Australia, showing a decreasing trend in total cholesterol and/or LHD-C with increasing age and use of lipid-lowering drugs [19–22]. However, investigations in countries such as Japan and India have shown an increasing trend in age-adjusted mean total cholesterol and triglycerides over the same period, despite an increase in the use of lipid-lowering drugs [23, 24]. In our study, females had higher cholesterol and triglyceride levels than males in the population aged 65 years and older, regardless of other factors. Compared to females, males had less hypercholesterolemia but more hypertriglyceridemia, with this difference using age 65 as the cutoff. In addition, smokers had higher total cholesterol, triglyceride, and LDL-C levels and lower HDL-C levels than nonsmokers. The prevalence of hypercholesterolemia and hypertriglyceridemia was significantly higher in smokers than in nonsmokers. Among them, smoking had the greatest effect on elevated triglyceride levels in Mexican-American participants, with a smaller effect in white participants and the least effect in black participants.

Many projects have researched the incidence and prevalence of hyperlipidemia, which is associated with many metabolic disorders in the clinic. Sparkar et al. suggested that hyperlipidemia is associated with an increased risk of nonalcoholic fatty liver disease in blacks and whites, affecting black adults in particular [25]. Among women with early breast cancer, hyperlipidemia was more prevalent in black women than in white women (28% versus 18%; *P* = 0.02) [26]. However, a national cross-sectional study in South Africa showed that black African men (0.64, 0.49–0.84) and women (0.52, 0.43–0.62) had significantly lower odds of hypercholesterolemia than white men and women, respectively [27]. Similarly, hypercholesterolemia was significantly higher in white patients with non-ST-segment elevation myocardial infarction than in patients of color (49% versus 34%, *P* < 0.001), including black, Asian, and ethnic minority patients [28]. In addition, among South African children and adolescents, other ethnic groups were more likely to have hypertriglyceridemia (1.94, 0.99–3.78) and low HDL-C (3.65, 2.33–5.72) than whites and blacks [29]. In another study, the prevalence of hypertriglyceridemia was found to be significantly lower in non-Hispanic black women than in non-Hispanic whites and Hispanics (5.1 versus 28.3 versus 30.5%, *P* < 0.01, respectively) [30]. The findings of the current studies are contradictory, and there is not a relatively consistent conclusion. Moreover, the current studies are limited to a specific population, and there is no analysis of racial differences in blood lipids in the general population. However, one thing is certain is that there are differences in the distribution of lipid levels in different subpopulations.

Our study suggests that lipid levels vary by race, sex, age, smoking, etc. In general, blacks have significantly lower lipid levels than other populations, and smokers have significantly higher lipids than nonsmokers. In addition, age also affects lipid levels, especially hormonal changes in women after menopause. Therefore, the diagnosis and treatment of hyperlipidemia should be done concerning the lipid levels of subpopulations, not the general population.

## Strengths and Limitations

Our study is the first to describe the distribution of blood lipids across ethnic, gender, and age groups. Besides, the prevalence of hypercholesteremia and hypertriglyceridemia across different ethnic, gender, and age were also explored. However, there were several limitations about this study. Our study did not include diet and medication use in the comparative analysis, and they may have influenced the lipid and study results. In addition, the data in this study were surveyed for the US population, and geographic characteristics such as climate and economy may have affected the results accordingly, so we may consider adding relevant lipid data from other geographic populations in the future to improve the accuracy of the data analysis.

## Conclusion

Distribution of population lipid levels and prevalence of hyperlipidemia based on differences in age, sex, race, and smoking status. Therefore, our analysis suggested a new view that the diagnosis and treatment of hyperlipidemia should be more accurate by referring to the lipid levels of subpopulations rather than those of the general population.

## Data Availability

The data that support the findings of this study are openly available in [NHANES] at [https://www.cdc.gov/nchs/nhanes/index.htm].

## Abbreviations

**HDL-C**: High-density lipoprotein cholesterol
**LDL**: Low-density lipoprotein cholesterol
